# A Hitherto Undescribed Disease of Childhood

**Published:** 1881-11

**Authors:** 


### A HITHERTO UNDESCRIBED DISEASE OF CHILDHOOD.

Antonio Rigo reports (*el Movimento Med.-Chir.*, *No.* 1, 1881) numerous cases of a peculiar affection which has been prevalent in Terra di Lavoro during the last sixty to seventy years. Its most characteristic feature consists in the formation of a pearl-gray, false membrane, of the size of a pea up to that of a centesimo, on the under surface of the tongue anywhere between the tip and frenulum. It is adherent to the mucous membrane, above the level of which it is but little prominent, and, on being torn off, while causing only slight hemorrhage, exposes an ulcerated base, which becomes covered with a new membrane in a day or two, despite cauterization with nitrate of silver. The rest of the mucous membrane of the mouth is not affected, there is no fœtor from the mouth, no swelling of the tongue or adjacent lymphatic glands, and no interference of nursing. The disease occurs only during the summer months, is always associated with intestinal catarrh, and is fatal in ninety per cent of the cases, death being due to inanition. The affection is confined to nurslings, occurring most frequently in the period immediately preceding the first dentition, and is not contagious, but infectious. It does not appear to be dependent upon any constitutional anomaly inherited from the parents, although these are usually country people living under the most impoverished conditions. The author has not met with the disease in its first stage, nor has he been able to make any post-mortem examinations or microscopical investigations of the false membrane. Its non-identity with diphtheria is established by the clinical features of the disease, and more particularly by the fact that diphtheritis has occurred in the district only since the last decennium.—*Allgem. Med. Central-Zeitung.*—*New York Medical Record.*

